# Effect of acupuncture on in vitro fertilization: An updated systematic review and data mining protocol

**DOI:** 10.1097/MD.0000000000010998

**Published:** 2018-06-15

**Authors:** Xiaotong Wang, Haixiong Lin, Mingzhu Chen, Jian Wang, Yuanlin Jin

**Affiliations:** a Shenzhen Bao’an Traditional Chinese Medicine Hospital Group, Guangzhou University of Chinese Medicine, Shenzhen; b The First School of Clinical Medicine; c Clinical Medical College of Acupuncture, Moxibustion and Rehabilitation, Guangzhou University of Chinese Medicine, Guangzhou, People's Republic of China.

**Keywords:** acupuncture, data-mining, in vitro fertilization, protocol, systematic review

## Abstract

**Background:**

Although many patients try to seek acupuncture to improve in vitro fertilization (IVF) outcomes, evidence regarding its efficacy and acupoints characters are lacking. The aim of this protocol is to evaluate the effectiveness and safety, as well as the acupoints characteristics of acupuncture in the treatment of female undergoing IVF, by conducting a systematic review and data mining.

**Methods:**

The following 6 databases will be searched from their inception to April 30, 2018: PubMed, Chinese National Knowledge Infrastructure, Wanfang, VIP database, Embase, and Cochrane Library. The randomized controlled trials (RCTs) or case–control studies of acupuncture that assess clinical effects and side effects in female undergoing IVF are included. The primary outcome measures will be number of oocytes retrieved, fertilization rate, oocyte cleavage rate, high-quality embryos rate, ovarian hyperstimulation syndrome (OHHS) incidence rate, clinical pregnancy rate (CPR), biochemical pregnancy rate (BPR), implantation rate, and cycle cancellation rate. Two reviewers will independently undertake data extraction and quality assessments. Data will be synthesized by RevMan V.5.3 software. Acupoints characteristics will be excavated using Traditional Chinese Medicine inheritance support system (TCMISS). Reporting bias will be assessed by Funnel plots, Begg test, and Egger test.

**Results:**

This review will assess the clinical efficacy and safety, as well as the acupoints characteristics of acupuncture on IVF.

**Conclusion:**

These findings will summarize the current evidence of acupuncture on IVF outcomes and may provide guidance for clinicians and infertile women to select acupuncture for IVF.

## Abstract 2

Supplemental Digital Content is available in the text

## 1 Introduction

Infertility is an important public health issue affecting approximately 7 million women of reproductive ages in the United States.^[1]^ In addition , about 6% of married women aged 15 to 44 years were unable to get pregnant after at least 12 consecutive months of trying to conceive.^[1]^ Infertility not only leads to instability in couples but also increases the feelings of depression, anxiety, and frustration.^[2,3]^ In vitro fertilization-embryo transfer (IVF-ET) may be the last possibility for many couples to get pregnant. Nevertheless, many IVF cycles do not lead to pregnancy, and repeated treatment cycles are required to achieve pregnancy.^[4]^ The repeated cycles not only increase the economic burden on families but also cost much time of the patients.^[5,6]^ Therefore, many patients also try to seek alternative therapies to improve reproductive outcomes, including acupuncture.^[7]^

Acupuncture, dates back at least 3000 years, is one of the most commonly used medical treatments in the world.^[8]^ Acupuncture has been utilized for gynecological and obstetrical diseases, and is one way of complementary and alternative medicine considered by infertile women seeking medical treatments.^[9,10]^ Its theory is based on the principles of meridians and acupoints of Chinese medicine, as well as the circulation of *qi* and blood.^[11,12]^ According to the theory of Chinese medicine, diseases are caused by the imbalance of *qi* and blood in the body, and could be alleviated by stimulating specific acupoints on the body surface.^[13,14]^ In recent years, many controversial results have been reported regarding the effect of acupuncture on IVF-ET outcomes. One meta-analysis conducted by El-Toukhy et al^[15]^ in 2008 showed that acupuncture around the time of ET has no difference in the CPR compared with sham acupuncture [risk ratio (RR) = 1.23, 95% confidence interval (95% CI) 0.96–1.58, *P* = .1]. In 2017, Qian et al^[11]^ conducted an updated meta-analysis with the aims to explore the clinical effect of different acupuncture methods in the Asian and non-Asian group, and found that electro-acupuncture could significantly improve the IVF outcomes, such as CPR [odds ratio (OR) = 1.81, 95% CI: 1.20–2.72, *P* = .005], BPR (OR = 1.84, 95% CI: 1.12–3.02, *P* = .02), live birth rate (LBR) (OR = 2.36, 95% CI: 1.44–3.88, *P* = .0007), and ongoing pregnancy rate (OPR) (OR = 1.94, 95% CI: 1.03–3.64, *P* = .04) in Asian group than those from the control groups. However, the previous review did not describe the different acupuncture methods on IVF outcome, and the latter one incorporated studies of different treatment cycles in the same subgroup, which may mislead clinicians and patients.

To our knowledge, no systematic review has been published to explore different acupuncture methods on IVF according to different treatment cycles yet. In addition, there is insufficient evidence to explore the acupoints characteristics of acupuncture on IVF. The purpose of our study is to explore the specific feasible methods of acupuncture on IVF and mining the acupoints characteristics using TCMISS.

## 2 Methods

This protocol was designed according to the Preferred Reporting Items for Systematic review and Meta-Analysis Protocols (PRISMA-P) (Supplementary File 1) and registered in PROSPERO (CRD 42018092543).

### 2.1 Study type

We will collect articles of RCTs or case–control studies that evaluated the therapeutic effects or side effects of acupuncture on IVF. There will be no restrictions regarding the race, region of the studies. Observational studies, case series, animal experiments, qualitative studies, comments, and reviews will not be included.

### 2.2 Participants

Women undergoing IVF with or without intracytoplasmic sperm injection (ICSI) treatment or ET will be included. There will not be any restrictions on age and original countries of the participants.

### 2.3 Interventions

Acupuncture intervention group will receive traditional acupuncture, electrical acupuncture, auricular acupuncture, paracervical block, or acupuncture sequential therapy. Control interventions, including no treatment, sham, or placebo acupuncture, will be included. The other treatments between intervention group and control group should be the same.

### 2.4 Outcome measures

For an eligible trial, it must have at least one of the following outcomes.

Primary outcome measures will be the number of oocytes retrieved, fertilization rate, oocyte cleavage rate, high-quality embryos rate, OHHS incidence rate, CPR, BPR, implantation rate, and cycle cancellation rate. Moreover, the criteria of data will be calculated on the basis of the follow requirements^[16,17]^: BPR is confirmed by a positive urine test or hCG serum 11 days after ET, and CPR is identified by ultrasound 4 to 6 weeks after ET that there have not less than one intrauterine gestational sac or fetal heartbeat.

Secondary outcome measures will be LBR, miscarriage rate, and side effects. The criteria of LBR is defined as a baby born alive after 24 weeks gestation.

### 2.5 Data sources

The following electronic databases will be searched from inception to April 30, 2018: The PubMed, Chinese National Knowledge Infrastructure, Wanfang, VIP database, Embase, and Cochrane Library. The free words or Mesh term search will contain the intervention methods, disease part, and study type: (“acupuncture” OR “acupuncture therapy” OR “electroacupuncture” OR “acupuncture points” OR “laser acupuncture” OR “auricular-acupuncture” OR “acupuncture ear” OR “moxibustion” OR “acupuncture analgesia”) and (“in vitro fertilization” OR “in vitro fertilizations” OR “test-tube fertilization” OR “test tube fertilization” OR “test-tube fertilizations” OR “fertilizations in vitro” OR “test-tube babies” OR “test tube babies” OR “test-tube baby” OR “assisted reproduction technology” OR “embryo transfer”) and (“randomized controlled trial” or “case control studies” or “trial”). The search strategy for PubMed is presented in Table 1. The equivalent search terms will be used in Chinese National Knowledge Infrastructure also shown in Supplementary File 2. Similar search strategies will be performed to the other databases. Reference lists of the potentially relevant articles will also be collected to discover additional clinical studies. We will include studies published in Chinese and English. Studies published in an abstract form will be excluded unless sufficient data could be attained from the abstract or authors. Besides, in order to ensure the reproducibility of treatment strategies, the strategies that are not repeated will be excluded.

**Table 1:**
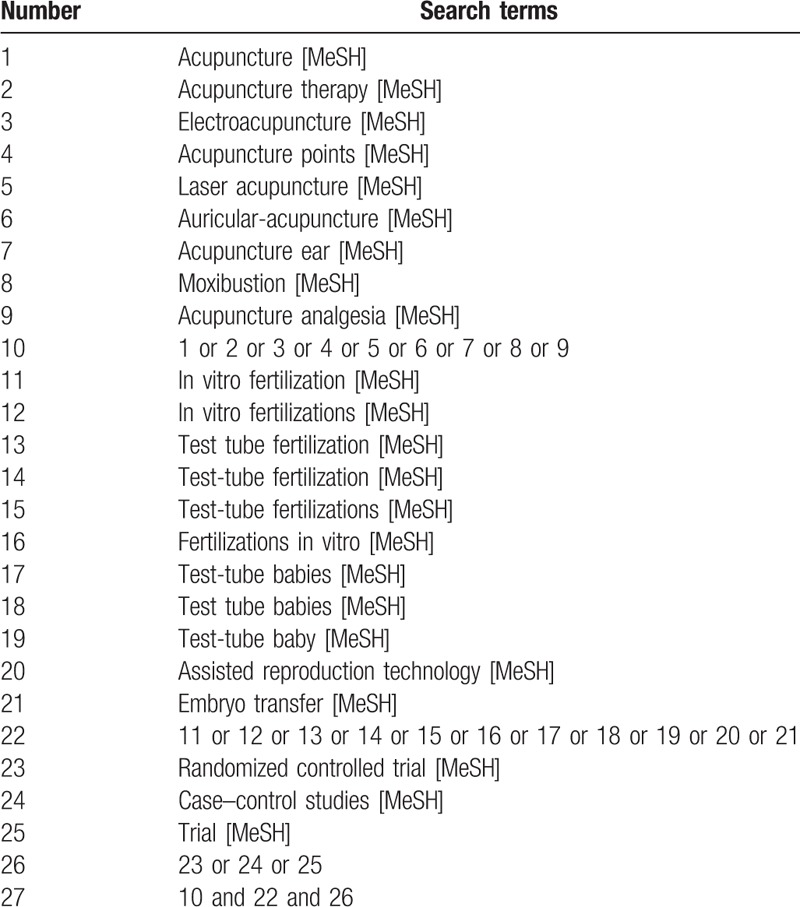
The search strategy for PubMed.

### 2.6 Study selection

**Figure 1:**
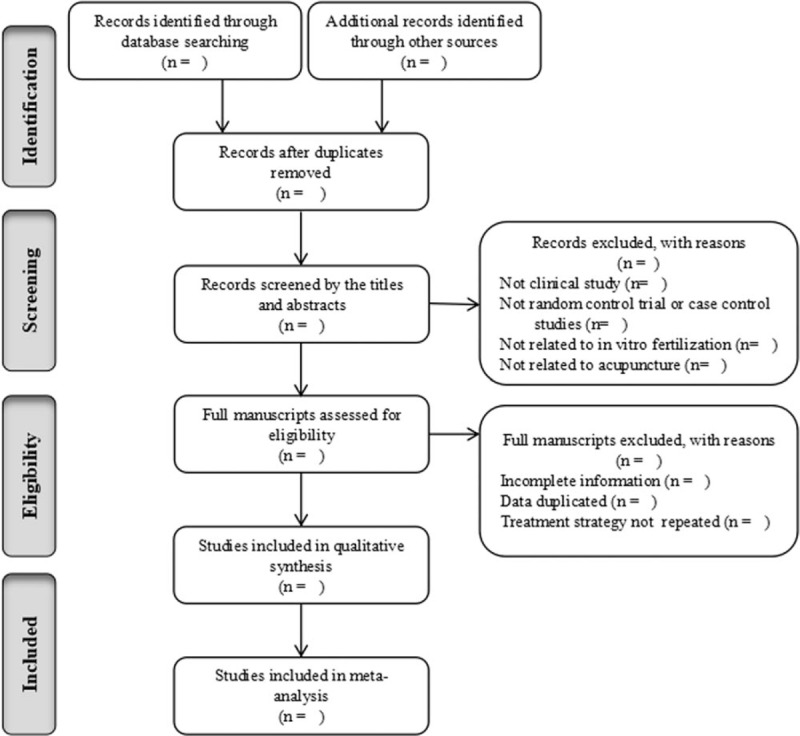
Flow chart of the search process.

All retrieved data will be imported into NoteExpress 3.2.0 and a duplicate article from different databases will be eliminated first. Two reviewer authors (Wang XT and Lin HX) will scrutinize the titles and abstracts independently, and then read full manuscripts for eligible trials according to the predefined selection criteria described above. Complete studies selection process will be documented and summarized in a PRISMA flow chart (http://www.prisma-statement.org/) (Fig. 1).

### 2.7 Data extraction and management

The following information will be extracted from all included studies by 2 independent reviewers (Wang XT and Lin HX) and saved in a data extraction sheet: first author names, publication year, area of the study, patients characteristics (gender, age, numbers of participants), relevant therapeutic method (intervention methods, frequency of treatment, and duration of treatment), dropout number, and clinical outcome from each included study. Any disagreements about inclusion will be resolved by consensus or arbitration by a third author (Yuanlin Jin).

### 2.8 Dealing with missing data

When there are insufficient or missing data, the original authors will be contacted to acquire additional data or clarification for unclear information via telephone or email. If unable to obtain missing data, the available data will be analyzed and we will pay attention to the potential impact of insufficient data on the results in the discussion section.

### 2.9 Risk of bias in included studies

The methodological quality of each eligible study will be evaluated using the risk of bias tool mentioned in the Cochrane handbook. The risk assessment covers 6 domains, including adequacy of randomization, concealment of allocation, blinding of outcome assessments, blinding of participants, integrity of outcome data, and selective reporting. What is more, the risk bias for each domain will be classified into 3 categories: low, unclear, or high risk. Especially, some clinical trials use the word randomization instead of randomization method. The risk of bias will be identified as high risk unless detailed randomization processes are mentioned.

### 2.10 Data synthesis and analysis

The statistical analyses will be performed using Review Manager V.5.3 software. Continuous data will be pooled and shown as mean differences or standardized mean difference with 95% CI. Dichotomous data will be pooled and presented as RR for with 95% CI. Heterogeneity among studies will be assessed using the Chi-square test and *I*^*2*^ values.^[18]^*I*^*2*^ ≤ 50% is considered no heterogeneity, and fixed effects model will be performed to analyze data.^[19]^*I*^2^ > 50% indicates mild or significant heterogeneity, and random-effect model will be conducted among the studies (*P* < .05, *I*^*2*^ > 50%).^[19]^

### 2.11 Additional analyses

Meta-regression and subgroup analysis will be carried out if substantial heterogeneity is identified. Sensitivity analysis will be considered, with the aims to ensure the robustness of the pooled result by removing low-quality trials. Subgroup analysis will be conducted according to different study characteristics, such as study location, study quality, intervention type, treatment duration. If the data extraction is insufficient, qualitative synthesis will be conducted instead of quantitative synthesis. Data mining will be used to analyze the acupoints characteristics of acupuncture for IVF by TCMISS.

### 2.12 Assessment of reporting biases

If the numbers of included studies are sufficient, the potential reporting bias will be evaluated by observing the symmetry of funnel plots. If asymmetry is detected by a visual inspection, Egger's test and Begg's test will be used to further assess the publication bias. Values of *P* > .05 in Begg's test or Egger's test indicate no significant bias.

### 2.13 Quality of evidence

The quality of the overall evidence will be assessed using the approach of the Grading of Recommendations Assessment, Development and Evaluation.^[20]^ The limitations of the clinical study, indirect evidence, inconsistencies, inaccuracies, and publication bias will be taken into account. The quality of the overall evidence will be classified into 4 categories: high, moderate, low, or very low.

## 3 Discussion

The average number of couples with fertility is 9% in the global.^[21]^ Many of them are seeking the services of human reproduction. However, nearly 75% of IVF cycles are unsuccessful.^[15]^ Acupuncture, an option of nondrug therapy, has been applied to regulate the female reproductive system.^[22]^ However, the impact of different acupuncture methods and the different roles they could play in IVF are still unclear. More and more clinical trials have been conducted to explore the clinical effect of different acupuncture methods on IVF. Some studies have revealed that women who received acupuncture during the IVF cycle have higher CPR.^[23,24]^ However, Gejervall et al found that electroacupuncture has no significant difference in pregnancy per transfer when compared with conventional analgesia (*P* = .470).^[25]^ To our knowledge, even though some relevant systematic reviews have been published to illustrate the effects of acupuncture on IVF,^[11,15]^ they did not strictly explore the clinical effects of different acupuncture methods on IVF according to different treatment cycles. Besides, many studies only report few clinical outcome, instead of multiple dimensions, to evaluate the clinical efficacy of acupuncture on IVF.^[26,27]^ Therefore, the purpose of this review is to assess the effect of different acupuncture on number of oocytes retrieved, fertilization rate, oocyte cleavage rate, high-quality embryos rate, OHHS incidence rate, CPR, BPR, implantation rate, cycle cancellation rate, LBR, miscarriage rate, and side effects of female undergoing IVF according to different treatment cycles. At the same time, intervention methods that are not repeated will not be included with the aims to ensure the accuracy and reliability of the results.

The data mining software TCMISS includes various functions, such as text mining, association rules analysis, and complex system entropy method.^[28]^ In recent years, it was applied to analyze the rules of acupoints in acupuncture treatment of disease and the composition rules of the prescriptions of distinguished traditional Chinese physicians, which will give feasible advice for the clinician.^[28,29]^ No protocols have been designed to assess the acupoints characteristics of acupuncture on IVF. The purpose of our study is to mining the acupoints characteristics using TCMISS.

Herein, this protocol will be the first to assess the clinical efficacy and safety, as well as the acupoints characteristics of acupuncture on IVF, and may benefit women undergoing IVF and practitioners in the fields of conventional medicine.

## Author contributions

This protocol was first conceived by Wang XT, with critical contributions from the other authors. Lin HX and Wang XT drafted the protocol and submitted the registration on PROSPERO. Jin YL revised the manuscript. Lin HX and Wang XT developed the search strategies and will conduct data collection and analyze independently. Chen MZ and Wang J will assess risk of bias. All authors contributed constructive comments on the paper and approved the final protocol.

**Data curation:** Xiaotong Wang, Haixiong Lin, Mingzhu Chen, Jian Wang.

**Investigation:** Xiaotong Wang.

**Methodology:** Xiaotong Wang, Haixiong Lin, Mingzhu Chen.

**Project administration:** Xiaotong Wang, Yuanlin Jin.

**Resources:** Xiaotong Wang, Haixiong Lin.

**Software:** Xiaotong Wang.

**Supervision:** Yuanlin Jin.

**Validation:** Jian Wang.

**Visualization:** Haixiong Lin.

**Writing – original draft:** Xiaotong Wang, Haixiong Lin.

**Writing – review & editing:** Xiaotong Wang, Haixiong Lin, Yuanlin Jin.

## Supplementary Material

Supplemental Digital Content
